# Bilateral Superior Gluteal Artery Perforator (SGAP) Flap: Modified Concept in Perineal Reconstruction

**DOI:** 10.3390/jcm13133825

**Published:** 2024-06-29

**Authors:** Maximilian Zaussinger, Gabriele Pommer, Katrin Freller, Manfred Schmidt, Georg M. Huemer

**Affiliations:** 1 Section of Plastic and Reconstructive Surgery, Kepler University Hospital, Krankenhausstrasse 9, 4020 Linz, Austria; m.zaussinger@hotmail.com (M.Z.); gabrielepommer@gmx.at (G.P.); katrin.freller@gmail.com (K.F.); 2 Medical Faculty, Johannes Kepler University Linz, Altenbergerstr. 69, 4040 Linz, Austria; office@drhuemer.com; 3 Doctoral Degree Program in Medical Science, Paracelsus Medical University, Strubergasse 21, 5020 Salzburg, Austria

**Keywords:** superior gluteal artery perforator (SGAP) flap, perineal reconstruction, perforator flap, abdominoperineal excision

## Abstract

**Background/Objectives:** Perineal reconstruction after abdominoperineal excision often requires complex closures and is fraught with wound healing complications. Flap-based approaches introduce non-irradiated vascularized tissue to the area of resection to fill a large soft-tissue defect and dead space, reduce the risk of infection, and facilitate wound healing. Employing perforator flaps with their beneficial donor site properties, the authors have developed a concept of bilateral superior gluteal artery perforator (SGAP) flaps to restore extensive perineal defects. **Methods**: This retrospective case series was conducted between September 2015 and December 2019. We included three patients who received bilateral SGAP flap reconstruction after oncological resection. One deepithelialized SGAP flap was used for obliteration of dead space, combined with the contralateral SGAP flap for superficial defect reconstruction and wound closure. **Results**: Within this patient population, two male and one female patient, with a median age of 62 years (range, 52–76 years), were included. Six pedicled SGAP flaps were performed with average flap dimensions of 9 × 20 cm (range 7–9 × 19 × 21). No flap loss or no local recurrence were documented. In one case, partial tip necrosis with prolonged serous drainage was observed, which was managed by surgical debridement. No further complications were detected. **Conclusions**: The combination of two SGAP flaps provides maximal soft tissue for defect reconstruction and obliteration of dead space, while maintaining a very inconspicuous donor site, even with bilateral harvesting. Given these advantages, the authors recommend this promising approach for successful reconstruction of perineal defects.

## 1. Introduction

Extended perineal soft tissue defects following abdominoperineal excision remain a challenging procedure in the field of reconstructive surgery. Besides achieving tumor-free oncological resection, the goals of reconstruction focus on stable skin and soft tissue coverage, obliteration of dead space, and restoration of pelvic anatomy [1,2]. Over the years, multiple approaches for perineal reconstruction have been described, primarily focusing on musculocutaneous flaps such as the vertically oriented rectus abdominis myocutaneous (VRAM) flap [3,4]. However, notable abdominal donor-site morbidity, including decreased abdominal strength and function after sacrificing of an abdominal muscle, represents a significant drawback of this invasive reconstructive approach [5]. Furthermore, harvesting the rectus abdominis muscle can weaken the abdominal wall, predisposing patients to hernia or bulge formation at the donor site. An alternative muscle flap is the utilization of the gracilis muscle [4]. For extensive or deep perineal defects, the gracilis muscle may not provide enough tissue volume and has a limited arc of rotation to achieve the desired coverage; hence, reliable reconstructive options are required.

Over the years, microsurgical techniques and a better understanding of vascular territories and their supplying vessels have evolved significantly, enhancing the precision and efficacy of reconstructive surgeries [6,7]. Consequently, the advent of constructing perforator-based flaps has provided a significant advancement in reconstructive treatment options and continues to expand as a cornerstone in modern reconstructive surgery [8,9]. One particularly versatile perforator flap from the buttock region is the superior gluteal artery perforator (SGAP) flap. The superior gluteal artery bifurcates into superficial and deep branches, with perforators emerging to supply the overlying skin and subcutaneous tissue. The SGAP flap utilizes these perforators, ensuring a robust blood supply while preserving the underlying muscle. First described by Koshima et al. in 1993, the SGAP flap has since been employed across various fields of reconstructive surgery, ranging from autologous breast reconstruction to lower extremity reconstruction [10,11,12,13]. This flap offers several advantages that make it particularly suitable for perineal reconstruction. One of the primary benefits is the sufficient tissue volume it can provide, which is essential for effectively filling large defects. Moreover, the preservation of abdominal wall integrity is a significant advantage of the SGAP flap, as it avoids the complications associated with muscle sacrifice and the opening of the abdominal cavity, which are common drawbacks of other reconstructive techniques such as the VRAM flap. The SGAP flap’s ability to provide ample tissue volume while maintaining the structural integrity of the donor site underscores its utility in the restoration of perineal defects [10,11,12,13]. By leveraging the reliable vascular supply from the superior gluteal artery perforators, surgeons can achieve robust and sustained tissue viability, which is critical for successful reconstruction and optimal healing outcomes. As a result, the SGAP flap has become an important tool in the armamentarium of reconstructive surgeons, offering a reliable and effective solution for complex perineal reconstructions and beyond [14].

In this report, we have developed a modified concept that should restore large and deep perineal defects following abdominoperineal excision using bilateral pedicled SGAP flaps. Surgical description and clinical cases demonstrate the reliability of this concept.

## 2. Materials and Methods

This retrospective case series was conducted in accordance with local ethical standards and with the Declaration of Helsinki for Ethical Treatment of Human Subjects. Written and verbal informed consent was obtained from all patients prior to their inclusion in the study. Due to the retrospective nature of the study and the small cohort size, formal Institutional Review Board (IRB) approval was not sought. The study period spanned from September 2015 to December 2019, during which three patients underwent perineal reconstruction using bilateral pedicled SGAP flap. Detailed analysis of patient demographics was performed, including age, sex, follow-up duration, and indications for the procedure. Additionally, surgical data were meticulously recorded, focusing on postoperative complications and outcomes. The inclusion criteria for the study were stringent, with general exclusion criteria encompassing heavy smoking, acute signs of infection, or medical contraindications such as the use of oral anticoagulants. These criteria were established to ensure the safety and appropriateness of the surgical intervention for each patient.

### Surgical Technique

**Figure 1:**
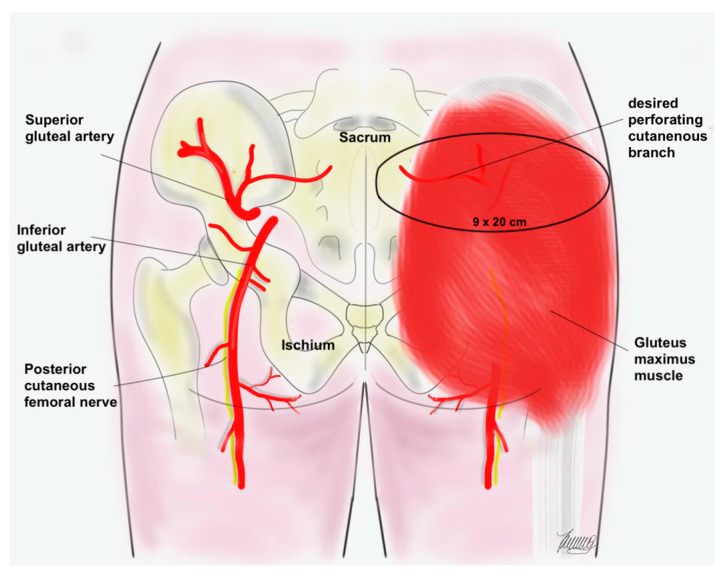
Illustration of the gluteal anatomy. The black circle demonstrates the vascular territory of the SGAP flap.

After abdominoperineal tumor excision, pelvic floor reconstruction was performed. In all three cases, a biological patch (Permacol, Medtronic, Minneapolis, MN, USA) was used. All SGAP flaps were harvested in the prone position. Preoperative Doppler ultrasonography verification of the supplying perforator was performed. According to current literature and our clinical experiences, the predicted location of the supplying perforator is 3 up to 6 cm lateral to the midline and within the upper third of the buttock [7]. Based on the location of the perforator, the skin island can be planned according to the individual patient’s localization of fat to be used to maximize volume with dimensions about 9 × 20 cm (Figure 1). The subcutaneous dissection can be beveled circumferentially to increase flap bulk if needed. After the gluteus maximus muscle fascia is opened, the dissection begins from lateral to medial to safely identify the perforators. Laterally, nondominant perforators are sacrificed and the medially dominant perforator is carefully dissected down through the gluteus maximus muscle fibers. The dissection continues down through the deep gluteal fascia until tension-free propeller-style rotation of the flap is possible. Emphasis is placed on the dissection of the pedicle of the future deep-located SGAP flap to allow sufficient and tension-free flap transposition. After deepithelialization of the deep SGAP flap, the flap is rotated into the defect to obliterate the dead space. After some deep fixation sutures, the first 15-gauge suction drain (Blake drain 15FR, Ethicon, Vienna, Austria) is placed. Finally, the contralateral SGAP flap is used for superficial defect reconstruction and wound closure. Before 3-layered wound closure, another 15-gauge suction drain (Blake drain 15FR, Ethicon, Vienna, Austria) is fitted. Patients were strictly instructed not to sit and lay on the donor site for at least three weeks after surgery. The standard postoperative management included antibiotic prophylaxis, anti-inflammatory therapy and low-molecular weight heparin (LOVENOX 4.000 IE (40 mg)/0.4 mL, Sanofi Winthrop Industrie, Le Trait, France) injections.

## 3. Results

In general, three patients (two male) underwent bilateral SGAP flaps for perineal reconstruction. Additionally, all patients received pelvic floor reconstruction with an acellular dermal matrix. The median age at the date of surgery was 62 years (range, 52–76 years), and the average follow-up time was 66 ± 26 months (range, 39–90 months). The average flap dimension was 9 × 20 cm (range 7–9 × 19–21). The mean stay in hospital was 10 days (range, 7–15). 

**Figure 2:**
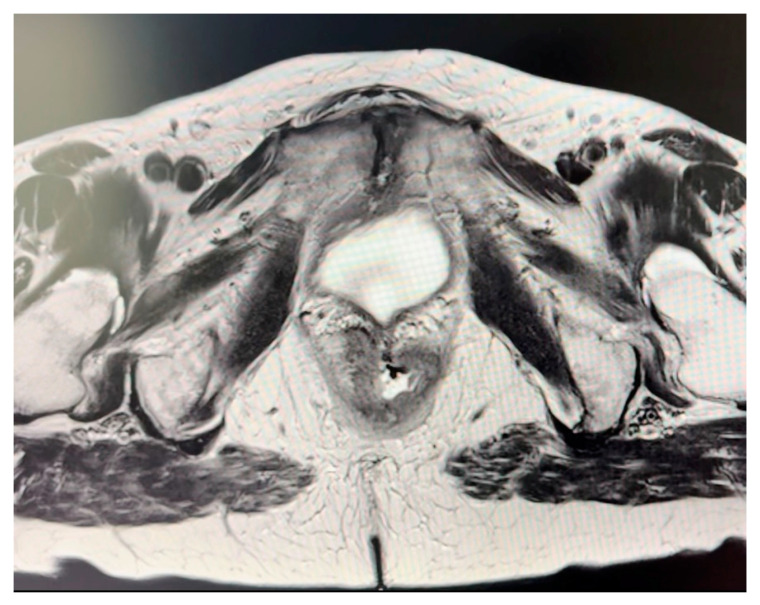
Magnetic resonance imaging (MRI) of the anal canal showing fistula formation into the vagina.

Indications for perineal reconstruction were invasive squamous anal carcinoma in two cases and adenocarcinoma of the vagina with infiltration of the anal canal in one case, as summarized in Table 1. The average size of resected tumor was 10.3 × 5.2 × 5.1 (range, 13.5–7.6 × 7–3.8 × 7.5–2.6) (Figure 2). No local recurrence was documented. Within this case series, one instance of partial tip necrosis of the deep flap with continuous wound discharge was observed and was salvaged with surgical debridement and secondary sutures. All donor incisions healed without complication.

### 3.1. Clinical Cases

#### 3.1.1. Case 1

**Figure 3:**
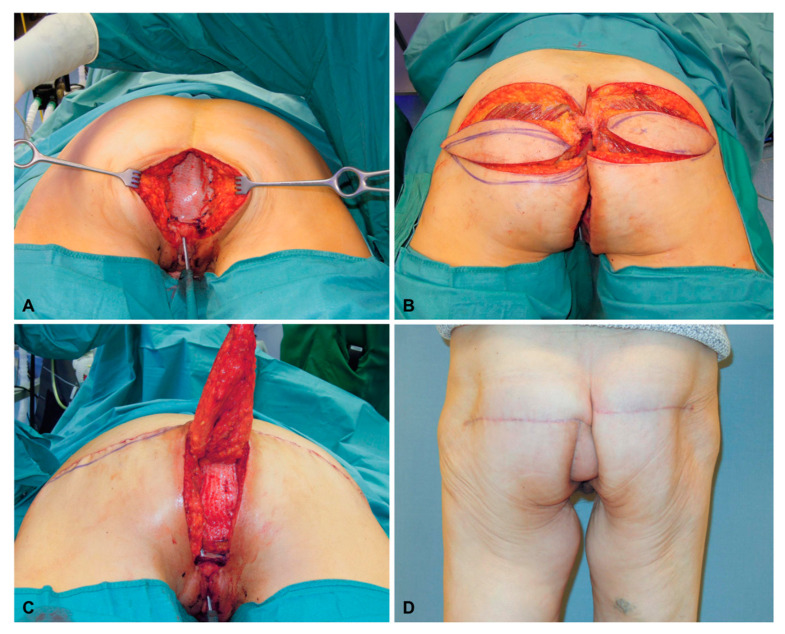
(**A**) After resection of an adenocarcinoma of the vagina with infiltration in the anal canal and placement of a biological patch for pelvic floor reconstruction. (**B**) Bilateral raised SGAP flaps before insertion (note: the cross marks the major perforator of the right flap). (**C**) After deepithelialization of the deep SGAP flap, the flap is rotated into the defect and covered by the superficial SGAP flap. (**D**) Six months after bilateral SGAP flap reconstruction.

A 62-year-old female patient underwent resection of an adenocarcinoma of the vagina with infiltration in the anal canal and biological patch for pelvic floor reconstruction (Figure 3A). Both flaps had dimensions of 8 × 20 cm (Figure 3B). The deepithelialized SGAP flap was used for the obliteration of dead space, combined with the contralateral SGAP flap for superficial defect reconstruction and wound closure (Figure 3C). Six months after reconstruction, the SGAP scars were very inconspicuous (Figure 3D).

#### 3.1.2. Case 2

**Figure 4:**
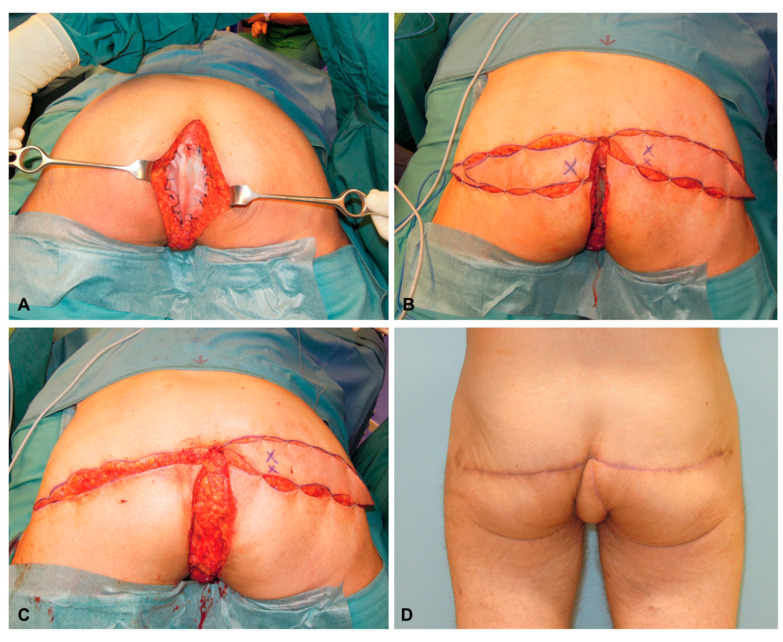
(**A**) After resection of an invasive squamous anal carcinoma and placement of a biological patch for pelvic floor reconstruction. (**B**) Bilateral raised SGAP flaps before insertion (Note: the cross marks the major perforator of the right flap). (**C**) The deep and deepithelialized SGAP flap is rotated into the defect for the obliteration of dead space. (**D**) Six months after bilateral SGAP flap reconstruction.

A 52-year-old male patient underwent resection of an invasive squamous anal carcinoma and received a biological patch for pelvic floor reconstruction (Figure 4A). Both flaps had dimensions of 9 × 21 cm (Figure 4B). After deepithelialization of the deep SGAP flap, the flap is rotated into the defect and covered by the superficial SGAP flap (Figure 4C). Six months after reconstruction the SGAP scars are very inconspicuous (Figure 4D).

## 4. Discussion

Several different methods have been described for perineal and vaginal reconstruction after abdominoperineal resection. Flap-based approaches introduce nonirradiated vascularized tissue to the area of resection to provide soft tissue coverage, eliminate dead space, and facilitate wound healing [15,16]. Unlike other frequently used techniques, the current literature on the safety and efficacy of perforator flaps for perineal reconstruction is heterogenous and limited. One useful and reliable perforator-based flap is the SGAP flap, which is already established for many other reconstructive purposes [11,12,17]. This gluteal perforator flap provides sufficient soft tissue and constant anatomy without sacrificing any muscle. Therefore, we have shifted our approach away from muscle-based flaps, such as the VRAM flap, towards this perforator-based flap in cases of perineal defects. These challenging defects after extensive resection often require large amounts of soft tissue that might not be provided using a single SGAP flap. As a result, we developed a modified reconstructive concept that includes two pedicled SGAP flaps: one deep and deepithelialized flap for the obliteration of dead space, covered by a contralateral SGAP flap for superficial wound closure. To our knowledge, this reconstructive concept for extended perineal defects has not been previously described. 

With regard to the literature, complication rates after abdominoperineal resection are high, around 50%, with infection being the most common complication [1,18]. Within this case series, the only noted complication was a partial tip necrosis of the deep flap, leading to prolonged wound discharge and wound dehiscence around the inferior border of the superficial SGAP flap. This complication occurred in a large, previously irradiated pelvic and perineal wound. To avoid such complications, innovative measures such as flap injections with platelet-rich plasma can improve wound healing and reduce complications [19]. No further complications were documented within our series. Apart from flap complications, the biological patch used for pelvic floor reconstruction poses a potential risk for further complications. Meticulous inset is essential, ensuring no gaps between fixation points to avoid herniation or obstruction of the bowel. Permacol, an acellular dermal matrix, is frequently utilized in such reconstructions due to its biocompatibility and ability to integrate with host tissues. However, its usage is not without challenges. Permacol can occasionally provoke a foreign body reaction, leading to chronic inflammation or infection [20,21]. Moreover, inadequate fixation of such a dermal matrix can result in patch migration or failure, emphasizing the necessity for precise surgical technique. Despite these risks, acellular dermal matrix provides a valuable option for pelvic floor reconstruction, particularly in complex cases where autologous tissue may not be viable. Using autologous tissue, such as the pedicled rectus abdominis muscle, is much more invasive and cannot preserve abdominal wall integrity [22]. Prior abdominal operations (e.g., hernia repair or laparotomy) or existing urostomy/ileostomy are further potential limitations of this autologous alternative. In such scenarios, the acellular dermal matrix offers a less invasive solution while still providing the necessary structural support for successful reconstruction.

Due to the characteristics of this specific patient population, which primarily consists of elderly individuals, those who are immunosuppressed, or those who have undergone irradiation, complications tend to be more frequent and multifactorial. The delicate balance of managing these complications is further complicated by the anatomical region involved, making the surgical outcomes more challenging. Therefore, assessing long-term outcomes, including donor site morbidity, becomes a crucial benchmark for successful perineal reconstruction. Our findings indicate that the SGAP flap is an excellent alternative to abdominally based flaps, primarily due to its lower donor site morbidity. This is particularly beneficial as the resulting scar is completely concealed by normal underwear, which significantly enhances patient satisfaction. Although the procedure necessitates a prone position, the preservation of the abdominal wall remains a major advantage of this approach. Unlike the traditional gold standard abdominal VRAM flap, the SGAP flap does not require transabdominal transposition, thereby preserving the integrity of the abdominal wall. Moreover, the SGAP flap is especially valuable for patients with existing urostomies or ileostomies, or those who have previously undergone abdominal flap procedures such as cosmetic abdominoplasty. In such cases, the VRAM flap might not be feasible due to the prior surgical alterations or the presence of stomas. Thus, the SGAP flap offers a viable and effective solution for perineal reconstruction in these complex situations, ensuring both functional and esthetic benefits while minimizing the risk of complications.

Based on our Doppler ultrasound and clinical findings, the superior gluteal vessels provide constant and well-suited perforators for the presented procedure. In general, regions of the body that are not beneficial for microvascular free tissue transfer due to missing or unfavorable recipient vessels benefit from reliable anatomy for local flap harvest. A further perforator-based flap from the gluteal region is the inferior gluteal artery perforator (IGAP) flap [23]. According to our experiences, the resulting scar tends to be more visible on the buttock and beyond the boundaries of the underwear. Another similar flap of this region represents the fasciocutaneous infragluteal (FCI) flap. Unlike the IGAP flap, the FCI flap is more caudally located and is supplied by the descending branch of the inferior gluteal artery, which is not defined as a perforator. Caution should be taken to avoid damage to the accompanying posterior cutaneous femoral nerve when performing flaps from the infragluteal region. Compared to the SGAP flap, potential nerve damage is almost impossible. We have thoroughly investigated the FCI flap and used it for perineal reconstruction as well [24,25]. However, the donor site of the FCI is closer to the genital region, which has some of the highest bacterial counts in the body and can therefore increase the risk of infection and wound breakdown. Additionally, the FCI donor site is more exposed for early sitting, which should not be overlooked in this specific patient population. 

## 5. Conclusions

In summary, the bilateral SGAP flap represents an excellent option for soft tissue reconstruction after extensive abdominoperineal resections. This new concept provides enough soft tissue for obliteration of the pelvic dead space and coverage of the perineal defect. Additionally, muscle sacrifice and opening of abdominal cavity are avoided. Furthermore, the constant presence of the SGAP perforators simplifies the surgical approach, resulting in a shorter surgical duration, which might improve patient recovery. While these data are promising, further investigations with larger case numbers are required. However, we believe that our presented results are promising and will contribute to improving outcomes within this patient population.

## Figures and Tables

**Table 1:** Demographical data and complications of patients who underwent bilateral SGAP flap reconstruction.

Patient	Age (yr)	Sex	Indication	Radiation Therapy to Defect Region	Time of Follow-Up (Months)	Complication
1	52	M	Invasive squamous anal carcinoma	No	90	No
2	76	M	Invasive squamous anal carcinoma	Yes	70	Tip necrosis (deep SGAP)
3	62	F	Adenocacrinoma of the vagina	No	39	No

F, female; M, male; yr, year.

## Data Availability

Data are contained within the article.
